# Diffusion tensor imaging of the brain in Pompe disease

**DOI:** 10.1007/s00415-022-11506-z

**Published:** 2022-12-08

**Authors:** Jan J. A. van den Dorpel, Marjolein H. G. Dremmen, Nadine A. M. E. van der Beek, Dimitris Rizopoulos, Pieter A. van Doorn, Ans T. van der Ploeg, Ryan L. Muetzel, Johanna M. P. van den Hout

**Affiliations:** 1 Center for Lysosomal and Metabolic Diseases, Department of Pediatrics, Erasmus MC University Medical Center Rotterdam, PO Box 2040, 3000 CA Rotterdam, The Netherlands; 2 Department of Radiology and Nuclear Medicine, Erasmus MC University Medical Center Rotterdam, Rotterdam, The Netherlands; 3 Center for Lysosomal and Metabolic Diseases, Department of Neurology, Erasmus MC University Medical Center Rotterdam, Rotterdam, The Netherlands; 4 Department of Biostatistics, Erasmus MC University Medical Center Rotterdam, Rotterdam, The Netherlands; 5 Department of Child and Adolescent Psychiatry/Psychology, Erasmus MC University Medical Center Rotterdam, Rotterdam, The Netherlands

**Keywords:** MRI, Diffusion tensor imaging (DTI), Pompe disease, Brain, White matter abnormalities

## Abstract

Enzyme replacement therapy has drastically changed prospects of patients with Pompe disease, a progressive metabolic myopathy. As classic infantile patients survive due to treatment, they exhibit progressive white matter abnormalities, while brain involvement in late-onset patients is not fully elucidated. To study the underlying microstructure of white matter, we acquired structural (T1, T2, FLAIR) and diffusion tensor imaging (DTI) of the brain in 12 classic infantile patients (age 5–20 years) and 18 late-onset Pompe patients (age 11–56 years). Structural images were scored according to a rating scale for classic infantile patients. Fractional anisotropy (FA) and mean diffusivity (MD) from classic infantile patients were compared to a reference population, using a Wilcoxon signed-rank, one sample test. Effect sizes (Hedges’ G) were used to compare DTI metrics across different tracts. For late-onset patients, results were compared to (reported) tractography data on normal aging. In classic infantile patients, we found a significant lower FA and higher MD (*p* < 0.01) compared to the reference population. Large-association fibers were most severely affected. Classic infantile patients with advanced white matter abnormalities on structural MRI showed the largest deviations from the reference population. FA and MD were similar for younger and older late-onset patients in large WM-association fibers. We conclude that, while no deviations from typical neurodevelopment were found in late-onset patients, classic infantile Pompe patients showed quantifiable, substantially altered white matter microstructure, which corresponded with disease stage on structural MRI. DTI holds promise to monitor therapy response in future therapies targeting the brain.

## Introduction

Pompe disease (glycogen storage disease type II, OMIM #232300) is a lysosomal storage disorder caused by a deficiency of the enzyme acid alpha-glucosidase (GAA), which leads to lysosomal glycogen accumulation mainly in muscle tissue, but also in the central nervous system [1]. Severe deficiency results in the classic infantile phenotype, which leads to death within the first year of life, if left untreated [2, 3]. A partial lack of enzyme activity is associated with the ‘late-onset’ phenotype, characterized by slowly progressive proximal muscle weakness and respiratory involvement [4].

Treatment with enzyme replacement therapy (ERT) using recombinant human acid alpha-glucosidase (alglucosidase alfa) has significantly improved prospects for patients with Pompe disease. In classic infantile patients, it led to increased—ventilator-free—survival, reversal of the cardiac hypertrophy and substantial improvements in muscle function, while in late-onset patients, muscle strength and pulmonary function stabilized [4–7].

Unfortunately, ERT does not cross the blood–brain barrier, hence leaving the glycogen accumulation in the brain untouched. Neuroimaging in surviving classic infantile patients revealed progressive white matter abnormalities, while neuropsychological assessment shows a decrease in processing speed, and sometimes more generalized cognitive decline [8–11]. In late-onset patients, reported brain involvement varies from no abnormalities to mild or moderate punctate white matter lesions, without evidence of general cognitive impairment [12–14].

These new insights show that for the treatment of patients with Pompe disease, the brain presents a new challenge. It is therefore important to better understand the extent and nature of these brain abnormalities and provide effect parameters for upcoming clinical trials with next-generation therapies, such as lentiviral gene therapy [15, 16], which include the brain as an additional target.

In this study, we used diffusion tensor imaging (DTI), an MRI technique that can provide quantitative information about white matter microstructure, to study the extent of brain involvement in Pompe disease.

DTI is based on water diffusion, which is determined, among other things, by the structure of axons and myelin. As the brain develops, changes in axonal configuration, packing, and myelination all contribute to more organized water diffusion (i.e., diffusion parallel, rather than perpendicular, to axons), leading to a higher fractional anisotropy (FA) and lower mean diffusivity (MD), two commonly derived DTI metrics [17].

Using DTI, we investigated (1) the microstructural properties of the cerebral white matter in patients with classic infantile and late-onset Pompe disease, (2) which white matter tracts were most severely affected, and (3) how DTI parameters correlated with white matter abnormalities (WMA) found on conventional MRI.

## Methods

### Patients

This cross-sectional study was conducted at the Center for Lysosomal and Metabolic Diseases, Erasmus MC University Medical Center in Rotterdam. This is the single referral center for Pompe disease in the Netherlands. Both classic infantile and late-onset patients were eligible for participation in the study. Classic infantile Pompe disease was defined as symptom onset before the age of 6 months, a hypertrophic cardiomyopathy, deficiency of alpha-glucosidase activity in leukocytes and/or fibroblasts, and two severe disease-causing GAA variants (http://www.pompevariantdatabase.nl); Late-onset Pompe disease was defined as onset of symptoms during childhood or adulthood, proximal or axial muscle weakness and/or respiratory muscle weakness, no cardiomyopathy, deficient enzyme activity in fibroblasts or leukocytes and two disease-causing variants in the GAA gene, with at least one mild or less severe GAA variant. Exclusion criteria were comorbidities or devices that did not permit MRI investigations. All patients who met the study criteria and visited our outpatient clinic on a regular basis were approached to participate in this study. All MR images were acquired between September 2018 and September 2019. The study protocol was approved by the institutional review board at the Erasmus Medical Center (MEC-2007-103; amendment 11). Written informed consent was provided by the patients and/or their caregivers.

### Reference sample

Reference DTI data from the Generation R Study [18], a population-based cohort consisting of 3050 children between 8 and 12 years old, were used for comparison.

### MRI acquisition and image processing

All data (including the reference sample) were acquired on the same 3 Tesla General Electric scanner (GE, MR750W, Milwaukee, WI) using an 8-channel head coil. Diffusion tensor MRI data were collected with 3 *b* = 0 volumes and 35 diffusion directions using an echo planar imaging sequence (TR /TE 12,500/72 ms; slice thickness 2 mm, FOV 240 × 240 mm, matrix = 120 × 120, number of slices = 65, Asset Acceleration Factor = 2, *b* = 900 s/mm^2^) [19]. Image preprocessing was conducted using FMRIB’s software library FSL [20]. Non-brain tissue was removed and diffusion tensor images were corrected for eddy current induced and motion artifacts [21]. Automated probabilistic tractography was conducted using the Autoptx plugin [22, 23]. A predefined set of seed and target masks, supplied by the AutoPtx software, were aligned to each participant’s diffusion data using a nonlinear registration. For the superior longitudinal fasciculus (SLF), inferior longitudinal fasciculus (ILF), inferior fronto-occipital fasciculus (IFO), superior thalamic radiation (STR), cortico-spinal tract (CST), posterior thalamic radiation (PTR) and anterior thalamic radiation (ATR), the FA and MD were calculated. Data quality assurance (RM, JD), consisted of a multistep process including visual inspection of sum of square error maps from the tensor fit, registration to standard space (FMRIB58 FA space) and inspection of probabilistic tractography output for accuracy [19, 24].

Structural MR images were collected using the following parameters: Sagittal 3D T2 FLAIR (TR/TE: 5500/148 ms; matrix 224 × 224), Axial T2 Propeller (TR/TE: 8507/100 ms; slice thickness 2 mm, matrix 320 × 320) and Coronal 3D T1 (TR/TE: 8.77/3.4 ms, slice thickness 1 mm; matrix 220 × 220). MR images were anonymized and independently scored by three observers (MD, HH, JD), and discrepancies were agreed upon in a consensus meeting which was led by pediatric neuro-radiologist (MD). For classic infantile patients, a previously published three-stage classification was used [8]. Stage 0: no brain involvement. Stage 1: white matter hyper-intensities limited to periventricular white matter and centrum semiovale. Stage 2: additional involvement of subcortical white matter, internal capsule, and external capsule. Stage 3: extension to involvement of u-fibers, basal ganglia, cortico-spinal tract, and/or infratentorial white matter. In late-onset patients, the Fazekas scale was used to quantify white matter abnormalities [25].

### Statistical analysis

All analyses were performed using the R Statistical Software (version 3.6.1). For bilateral tracts, left and right values were averaged (weighted by tract volume) to a yield a single DTI metric per tract. We compared the results obtained in classic infantile patients to the reference sample, using a Wilcoxon signed-rank one sample test, using the mean of the reference sample as the population mean. Multiple testing correction was applied using FDR (7 tracts; 2 DTI metrics). Effect size (Hedges’ G) was calculated and used to compare DTI metrics across different tract.

The lack of reference data for older individuals on the MRI scanner used in our study prompted us to use a descriptive approach to compare late-onset patients with tractography data on normal aging as reported in the literature [26, 27].

## Results

### Patient characteristics

In this study, 12 classic infantile patients and 18 late-onset patients were included (Table 1). The age at time of evaluation of the classic infantile patients ranged from 5.0 to 20.0 years. All classic infantile patients presented symptoms shortly after birth (range 0.0–0.5 years) and were subsequently treated with ERT (median ERT duration 7.1 years, range 4.9–19.6 years). Two of the classic infantile patients were CRIM-negative. The late-onset patients were between 10.5 and 55.5 years of age, with a median disease duration of 18.7 years (8.1–28.8). Three late-onset patients, who had been diagnosed because of an affected sibling or were analyzed after finding of hyperCKemia during routine blood testing, did not have any muscle involvement at the time of assessment, and did not receive ERT. All symptomatic late-onset patients were treated with ERT, with a median duration of 10.2 years (3.9–14.3).

**Table 1:** Patient characteristics and radiological findings

Pat no	Phenotype	GAA variant	CRIM status	Enzyme activity^§^	Age at symptom onset(yrs)	Age at start ERT (yrs)*	Functional status	Current age (yrs)	T2 MRI stage
1	Classic infantile	c.525del c.525del	−	0.3	0.1	0.2	Walking	5.0	2
2	Classic infantile	c.2481 + 102_2646 + 31del538 c.2481 + 102_2646 + 31del538	+	0.0	0.0	0.3	Walking	5.3	1
3	Classic infantile	c.525del c.2481 + 102_2646 + 31del538	+	0.4	0.1	0.3	Wheelchair (partial)	5.8	2
4	Classic infantile	c.1551 + 1G > A c.1551 + 1G > A	+	0.1	0.2	0.4	Walking	6.0	3
5	Classic infantile	c.525del c.525del	−	0.3	0.1	0.5	Wheelchair (partial)	6.5	2
6	Classic infantile	c.925G > A c.2608C > T	+	0.4	0.4	0.4	Wheelchair (partial)	6.8	3
7	Classic infantile	c.525del c.1933G > A	+	0.2	0.0	0.0	Walking	8.0	0
8	Classic infantile	c.2481 + 102_2646 + 31del538 c.525del	+	0.4	0.0	0.3	Walking	8.0	3
9	Classic infantile	c.2481 + 102_2646 + 31del538 c.2481 + 102_2646 + 31del538	+	0.2	0.0	0.2	Wheelchair (dependent)	10.0	2
10	Classic infantile	c.525del c.2481 + 102_2646 + 31del538	+	0.2	0.1	0.2	Wheelchair (dependent)	11.0	2
11	Classic infantile	c.2481 + 102_2646 + 31del538 c.2481 + 102_2646 + 31del538	+	0.3	0.0	0.0	Walking	13.9	3
12	Classic infantile	c.2481 + 102_2646 + 31del538 c.1799G > A	+	0.6	0.0	0.3	Wheelchair (dependent)	20.0	3
13	Late onset	c.-32-13 T > G c.525del	+	NA	AS	–	Walking	10.5	–
14	Late onset	c.-32-13 T > G c.2135 T > C	+	NA	0.8	1.1	Walking	11.3	–
15^@^	Late onset	c.-32-13 T > G c.525del	+	8.7	8.9	10.5	Walking	17.0	–
16^@^	Late onset	c.-32-13 T > G c.525del	+	NA	AS	–	Walking	19.7	–
17	Late onset	c.1643C > T c.2481 + 102_2646 + 31del	+	NA	2.7	6.0	Walking	20.2	–
18^%^	Late onset	c.-32-13 T > G c.525del	+	11.9	5.0	11.0	Walking	20.7	–
19^%^	Late onset	c.-32-13 T > G c.525del	+	NA	AS	–	Walking	22.9	–
20	Late onset	c.-32-13 T > G c.1933G > A	+	6.2	13.0	14.3	Walking	26.5	–
21	Late onset	c.-32-13 T > G c.379_380delTG	+	18.0	28.3	33.5**	Wheelchair (partial)	43.3	–
22	Late onset	c.-32-13 T > G c.172C > T	+	12.7	22.6	30.3	Cane	43.9	–
23	Late onset	c.-32-13 T > G c.525del	+	10.2	22.8	31.5	Walking	44.5	–
24	Late onset	c.-32-13 T > G c.2608C > T	+	8.8	17.3	41.2	Walking	45.2	–
25	Late onset	c.-32-13 T > G c.2481 + 102_2646 + 31del	+	14.0	24.4	35.5	Walking	47.1	–
26	Late onset	c.-32-13 T > G 1548G > A	+	11.0	29.7	39.9	Walking	49.5	–
27	Late onset	c.-32-13 T > G c.525del	+	4.2	21.0	45.9	Walking	49.8	–
28	Late onset	c.-32-13 T > G c.525del	+	17.0	31.9	38.9	Walking	49.8	–
29	Late onset	c.-32-13 T > G c.525del	+	9.7	27.1	44.7	Wheelchair (partial)	50.8	–
30	Late onset	c.-32-13 T > G c.525del	+	18.0	36.8	44.2	Wheelchair (partial)	55.5	–

*Yrs* years, *AS* asymptomatic, *ERT* enzyme replacement therapy

^§^Residual alpha-glucosidase enzyme activity, measured in fibroblasts, *The dose for pts 1–9 was all 40 mg/kg/week from start. For pts 10–12, the dose was elevated from 20 mg/kg/2 weeks to 40 mg/kg/week, pts 13–30 were treated with 20 mg/kg/2 weeks, NA Not available, ^@,%^Siblings, two still asymptomatic, both diagnosed after sibling, **ERT was discontinued at age 39.5 years, at request of patient

### Microstructural white matter differences in patients with classic infantile Pompe disease

We used DTI tractography data to gain more insight into the microstructural properties of cerebral white mater in classic infantile Pompe disease.

One of the tracts that is of particular interest is the SLF, which consists of association fibers, forming important connections between the frontal and parietal cortices. This is an area where marked abnormalities are seen at an early stage on conventional MRI (T2-weighted, FLAIR) in patients with classic infantile Pompe disease [8].

**Fig. 1:**
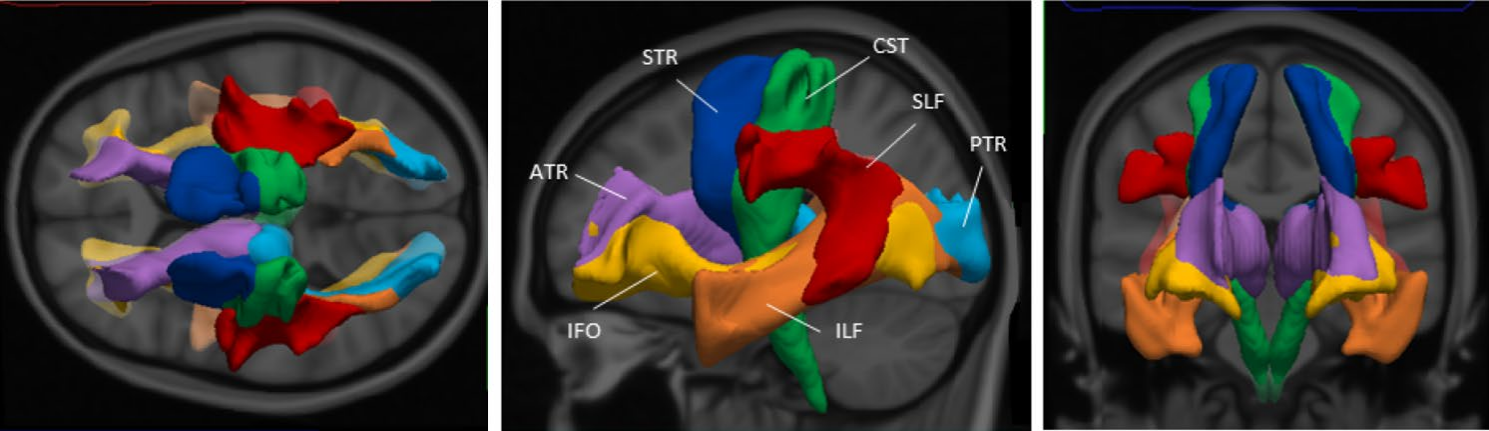
Visual representation of reconstructed white matter tracts. ATR anterior thalamic radiation, *CST* cortico-spinal tract, *IFO* inferior fronto-occipital fasciculus, *ILF* inferior longitudinal fasciculus, *PTR* posterior thalamic radiation, *SLF* superior longitudinal fasciculus, *STR* superior thalamic radiation

We found a significantly lower FA and higher MD (both *p* < 0.01) of the SLF in our cohort of classic infantile Pompe patients compared to the reference group (Fig. 1, Table 2), with a large effect size (Hedges G’. FA = 6.6, MD = − 11.5).

**Table 2:** Tractography results in patients with classic infantile Pompe disease and healthy controls

WM Tract#	Group	FA		MD	
		Median (range)	Effect size	Median (range)	Effect size
SLF	Patient	0.24 (0.19–0.40)	6.62	1.08 (0.78–1.25)	− 11.52
	Control	0.40 (0.33–0.46)		0.78 (0.71–0.85)	
ILF	Patient	0.33 (0.27–0.42)	4.85	0.98 (0.87–1.04)	− 5.18
	Control	0.43 (0.37–0.49)		0.84 (0.75–0.92)	
IFO	Patient	0.36 (0.28–0.44)	4.51	1.03 (0.85–1.24)	− 8.58
	Control	0.46 (0.39–0.53)		0.82 (0.75–0.89)	
STR	Patient	0.33 (0.30–0.42)	4.21	0.92 (0.80–0.97)	− 5.98
	Control	0.40 (0.35–0.45)		0.78 (0.72–0.84)	
CST	Patient	0.49 (0.45–0.54)	2.31	0.92 (0.79–0.96)	− 3.24
	Control	0.54 (0.48–0.60)		0.78 (0.67–0.89)	
PTR	Patient	0.38 (0.33–0.43)	2.09	0.99 (0.83–1.07)	− 4.66
	Control	0.42 (0.36–0.48)		0.82 (0.72–0.92)	
ATR	Patient	0.35 (0.33–0.39)	1.12	0.93 (0.82–0.98)	− 4.48
	Control	0.37 (0.32–0.42)		0.82 (0.75–0.89)	

^#^Difference between patient and control group was significant (*p* < 0.01, Wilcoxon one sample test) for all tracts

*FA* fractional anisotropy, *MD* mean diffusivity (10^–3^ mm^2^/s), *WM* white matter, *SLF* superior longitudinal fasciculus, *ILF* inferior longitudinal fasciculus, *IFO* inferior fronto-occipital fasciculus, *STR* superior thalamic radiation, *CST* cortico-spinal tract, *PTR* posterior thalamic radiation, *ATR* anterior thalamic radiation

Anatomically, in contrast to the SLF, the PTR traverses through a relatively unaffected area as indexed by conventional MRI (T2-weighted, FLAIR). Group-level comparison of classic infantile patients and the reference group, showed a significantly lower FA and higher MD (*p* < 0.01), (Fig. 1, Table 2). However, the effect size (Hedges’ G) of these differences was smaller (FA 2.1, MD − 4.7) compared to the SLF.

### Pattern of microstructural white matter differences in patients with classic infantile Pompe disease

To further elucidate the pattern in which microstructural changes reflected by DTI metrics occur in different parts of the brain, we explored five other white matter tracts throughout the brain.

We found a significant difference (*p* < 0.01) for all studied white matter tracts (Table 2, Fig. 1), with a lower FA and higher MD in patients compared to the reference population. Large-association tracts, such as the SLF, ILF and IFO, showed the largest differences in FA and MD between patients and controls, as reflected by Hedges G’ effect size. A smaller effect size was found for CST, as well as the thalamic radiations, such as the STR, PTR and ATR.

### Correlation of DTI and structural MRI in classic infantile patients

Next, we studied whether differences in DTI outcome parameters of individual classic infantile patients were related to conventional (T2-weighted) MRI findings. One patient (age 8.0), showed no abnormalities on T2-weighted MRI, one patient had stage 1 (age 5.3 years), five patients stage 2 (median age 6.5 years, range 5.0–11.0 years), and five patients stage 3 abnormalities (median age 8.0 years, range 6.0–20.0 years).

**Fig. 2:**
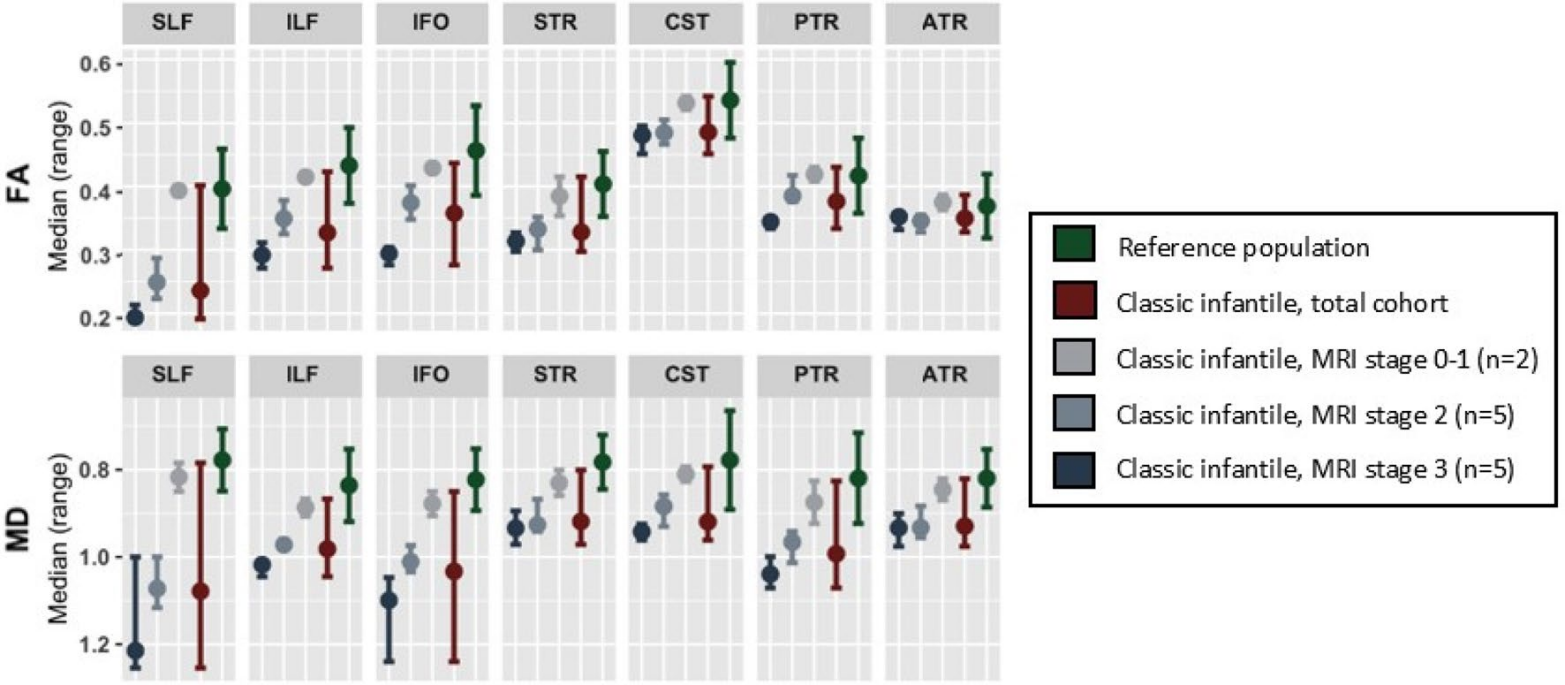
Tractography results of classic infantile patients and reference population. White matter tracts are sorted from left to right by effect size of FA. White matter tracts with a large effect size show larger differences between MRI stages. *FA* fractional anisotropy, *MD* medial diffusivity (*10^−3^mm^2^/s), *SLF* superior longitudinal fasciculus, *ILF* inferior longitudinal fasciculus, IFO inferior fronto-occipital fasciculus, *CS*T cortico-spinal tract, *PT*R posterior thalamic radiation, *ATR* anterior thalamic radiation

In patients without or with only mild T2 abnormalities (stage 0 or 1), FA values were within the reference range, while for patients with moderate or severe white matter abnormalities (stage 2 and 3), FA values were markedly lower. In general, patients with advanced white matter abnormalities (stage 3) on structural MRI, consistently showed the largest deviations in white matter microstructural metrics, compared to the reference sample (Fig. 2).

Furthermore, in the white matter tracts with the largest deviations (i.e., largest Hedges G’ effect size) compared to the reference group (i.e., the SLF, ILF and IFO), the differences in FA or MD were larger between the MR stages, pointing at an ongoing process of further derailment from normal for the most severely affected tracts (Fig. 2). For example, for the SLF (FA reference range 0.33–0.46), median FA was 0.39 (range 0.39–0.40) for stage 1, 0.25 (range 0.22–0.29) for stage 2 and 0.19 (range 0.19–0.21) for stage 3, while for the PTR, the differences between the stages were less apparent (FA reference range 0.36–0.48), with a median FA of 0.42 (range 0.41–0.43) for stage 1, 0.39 (range 0.38–0.42) for stage 2 and 0.34 (range 0.33–0.35) for stage 3.

### Microstructural properties of cerebral white matter in patients with late-onset Pompe disease related to age

Most patients with late-onset Pompe disease (13/18) had a completely normal structural MRI (T2w, FLAIR). Three patients (pat. 13, 16 and 29) had one or two a-specific focal punctate WMA, while two patients (pat 21 and 27) had slightly more extensive WMA (Fazekas score 1 or 2), but none of the patients had the extensive white matter lesions typical for the classic infantile phenotype [14].

Since the SLF, ILF and IFO appeared to be particularly involved in classic infantile Pompe patients, we assumed that the greatest chance of finding—early—abnormalities was within these tracts. We examined FA and MD for these tracts in patients with various ages.

**Fig. 3:**
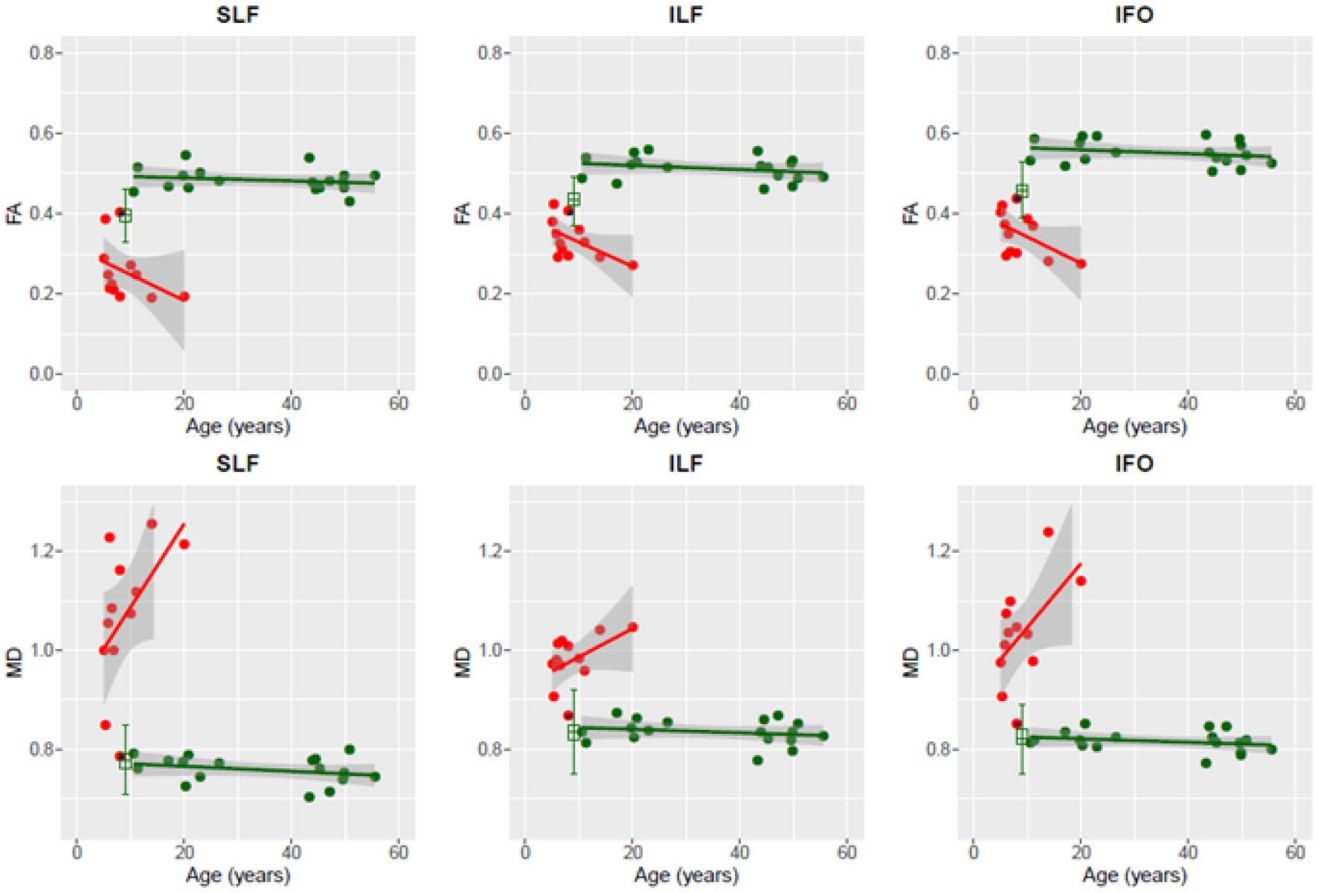
Tractography in late-onset and classic infantile Pompe patients. Green: late-onset Pompe patients; red: classic infantile Pompe patients; red with blue dot: classic infantile Pompe patient without abnormalities on structural MRI; green boxplot: reference sample. *FA* fractional anisotropy; *MD* mean diffusivity, *SLF* superior longitudinal fasciculus, *ILF* inferior longitudinal fasciculus, *IFO* inferior fronto-occipital fasciculus

Comparison of FA and MD values of younger (age range 19.7–26.5 years) and older (age range 43.3–55.5 years) adult patients for the three white matter tracts showed values in the same range; as shown for SLF, (median FA value 0.49 (range 0.46–0.55) for the younger patients against 0.48 (range 0.43–0.54) for the older group; median MD (10^–3^ mm^2^/s) value 0.77 (range 0.72–0.79) against 0.75 (range 0.70–0.80)); the ILF, (median FA 0.53 (range 0.51–0.56) against 0.51 (range 0.46–0.55); median MD (10^–3^ mm^2^/s) 0.84 (range 0.82–0.86) against 0.83 (range 0.78–0.87)); and IFO (median FA 0.58 (range 0.54–0.59) against 0.54 (range 0.50–0.59); median MD (10^–3^ mm^2^/s) 0.82 (0.80–0.85) against 0.81 (0.77–0.85)) (Fig. 3). This is a similar pattern as to what is found in literature during adulthood (29).

## Discussion

Since there is increasing evidence that white matter abnormalities in the brain and suboptimal cognitive functioning are part of the clinical phenotype in long-term surviving classic infantile Pompe patients, we sought to investigate the underlying microstructural properties, involvement across different white matter tracts and relation between those microstructural abnormalities and white matter abnormalities observed on structural MRI.

We found that patients with classic infantile Pompe disease had lower FA and higher MD compared to the reference group, indicating that white matter microstructure is disrupted [17, 28]. This may be due to myelin breakdown, glycogen accumulation, inflammation and gliosis.

The association between white matter hyper-intensities, and combined decreased FA values with increased MD values has been described in multiple diseases [29–31]. As well as more specifically, in two other lysosomal storage disorders, metachromatic leukodystrophy (MLD) and Krabbe disease, which both present with white matter abnormalities. In these diseases, DTI also shows a decrease of FA and an increase in MD [32, 33]. It is postulated that oligodendrocyte dysfunction subsequently led to loss of myelin (MLD) [34, 35] or demyelination and reduced myelination (Krabbe) [36], which together with other processes, such as substrate accumulation and inflammation, resulted in the microstructure changes as shown by the DTI.

Autopsy studies in classic Infantile Pompe disease are limited to patients deceased at young age. Nevertheless, glycogen accumulation has been described in the brain, especially in astrocytes, with oligodendrocytes being relatively spared [37, 38]. Oligodendrocytes being relatively spared in Pompe disease, could explain why, during the first years of life, myelination is only mildly delayed on structural brain imaging, and why most patients show normal development during early childhood.

Second, we found that we can quantify further deterioration in affected white matter using DTI as an objective measure. This offers an advantage over structural MRI which does not provide a quantitative measure of white matter damage. We observed that DTI outcome parameters reflected disease progression on structural MRI. With progression of structural abnormalities to different areas of the brain on T2w MRI, areas that are affected early in the disease course show further deterioration. This was particularly evident in association tracts; in these early affected tracts, we found the most apparent changes in diffusion parameters, particularly in stage 3 patients.

Third, we found that the large WM-association tracts (SLF, ILF, IFO), forming connections between frontal, parietal, temporal and occipital lobes, were the most severely affected tracts. Interestingly, studies in multiple sclerosis have shown that abnormalities in these tracts are associated with a lower processing speed [39]. A decrease in processing speed is one of the earliest and most prominent neurocognitive symptom in patients with classic infantile Pompe disease [8, 40].

Finally, in the brain of late-onset patients, we found a stable age-related FA and MD. With some caution, due to lack of a matched reference population, we consider these findings similar to the stage of relative stability found during normal aging. During normal development and aging, we can distinguish a 3-stage trajectory of FA and MD: first, during childhood, a sharp developmental increase in FA and reduction in MD is observed, followed by a period of relative stability during mid-adulthood, with a decrease in FA and increase in MD in senescence [26, 27]. Our findings are in line with previous reports using structural MRI, which found that there is no evidence for apparent brain involvement in late-onset Pompe disease [13, 14]. This is potentially explained by residual enzyme activity of 10–20% in late-onset patients, which may be sufficient to prevent accumulation of glycogen in neuronal cells in these patients.

Our study had several limitations: (1) the sample size was relatively small; however, considering the rarity of the disease, we were still able to include a representative cohort with a substantial number of patients, (2) we lacked an age-matched reference sample for the older patients. Despite these limitations, given the large effect sizes for FA and MD in classic infantile patients compared to the reference cohort, we consider our results to be robust.

While structural MRI studies, remain most important for pattern description and combining (multicenter) data, our study shows that DTI provides significant added value. First, to objectively quantify the progression of white matter abnormalities, which is important in light of new emerging therapies targeting the brain [15, 16], as well as for follow-up studies defining variables that explain differences in brain involvement between patients. Second, combining DTI and structural MRI, the use of DTI in the study of white matter, which is still appearing normal (on structural MRI), could potentially capture early changes and thus provide valuable information on the onset of microstructural changes.

## Conclusion

While in late-onset patients, no DTI abnormalities were found, brain white matter microstructure is substantially altered in classic infantile Pompe disease. This indicates disruption of myelinated axons, the origin of which needs further study. DTI alterations in classic infantile patients, corresponded with the disease stage rated on structural MRI. Long-association tracts appeared to be involved most. This study indicates that DTI holds promise and deserves further study as a potential parameter to monitor the effect of emerging treatments targeting the brain.

## Data Availability

Anonymized data can be made available to qualified investigators on request.
